# Lateral supracerebellar infratentorial approach for a ruptured cerebellomesencephalic fissure arteriovenous malformation resection

**DOI:** 10.3171/2020.10.FOCVID2090

**Published:** 2021-01-01

**Authors:** Livio Pereira, Eduardo Vieira

**Affiliations:** Department of Neurological Surgery, Hospital Da Restauração, Recife, Brazil

**Keywords:** arteriovenous malformation, cerebellomesencephalic fissure, posterior fossa

## Abstract

In this surgical video, the authors present a case of a 24-year-old male patient who presented with sudden-onset headache and imbalance. On examination, he had a right-sided dysmetria and was otherwise neurologically intact. MRI showed a right cerebellar hematoma associated with multiple flow voids in the cerebellomesencephalic fissure and an enlarged lateral mesencephalic vein. Preoperative angiogram confirmed an arteriovenous malformation supplied by branches of the superior cerebellar artery. The patient underwent a lateral supracerebellar infratentorial approach for resection of the arteriovenous malformation (AVM). He recovered well from surgery and was discharged home on postoperative day 6. Postoperative angiogram confirmed complete AVM resection.

The video can be found here: https://youtu.be/tY4Go2n7V80

## Transcript

**0:22** This video will demonstrate the lateral supracerebellar infratentorial approach for a ruptured cerebellomesencephalic fissure arteriovenous malformation (AVM) resection.

**0:31** The patient is a 24-year-old man who presented with a sudden-onset headache and imbalance. On exam, he had a right dysmetria and was otherwise neurologically intact.

**0:41** Here is his MRI imaging studies that demonstrates this right-sided intracerebellar hemorrhage, which was treated conservatively.

**0:53** We can also see an enlarged lateral mesencephalic vein and multiple flow voids within the cerebellomesencephalic fissure.

**1:02** The preoperative angiogram demonstrated a somewhat diffuse arteriovenous malformation supplied by branches of the right superior cerebellar artery. The AVM drainage was through an enlarged lateral mesencephalic vein up to the basal vein of Rosenthal. The patient’s Spetzler-Martin grade was III, with 1 point each for size, eloquence, and deep venous drainage.^1^

**1:31** Approximately 6 weeks after hemorrhage, the patient underwent surgery for resection. He was placed in a three-fourths prone position with neck flexion. A small right lateral suboccipital craniotomy was performed, and the transverse sinus was exposed. The dura mater was opened and retracted superiorly in order to elevate the transverse sinus and expand the supracerebellar infratentorial operative corridor.^2–4^

**1:59** The dissection begins with coagulation and division of these small bridging veins from the cerebellum to the tentorium.

**2:14** The dissection proceeds deeper until we reach the ambiens cistern. We can now see the fourth nerve beneath the arachnoid membrane.

**2:24** The trochlear nerve is now dissected, protected, and mobilized superiorly.^5^

**2:46** The dissection proceeds laterally until the trunk of the superior cerebellar artery (SCA) is found. It is then followed distally until the bifurcation of the SCA is visualized.

**2:59** Here we can see the bifurcation of the SCA into the superior and inferior branches.

**3:08** The inferior branch is followed distally and here we can see that it dives into the cerebellomesencephalic fissure to supply the AVM. On the other hand, the superior branch runs alongside the nidus but does not emit feeding arteries to the AVM. Underneath both arteries, we can see the arterialized lateral mesencephalic draining vein.

**3:30** A temporary clip is placed in the inferior division of the superior cerebellar artery, and the vessel is now coagulated and divided.

**3:57** In order to reach the deepest portion of the cerebellomesencephalic fissure, an incision is made in the cerebellum and the initial circumdissection of the nidus is carried out beneath the AVM.^6^

**4:25** The dissection continues until we reach the brainstem. Here we can see a portion of the nidus in close contact with the root of the trigeminal nerve. This part of the nidus is coagulated so that it shrinks and then it is dissected free of the brainstem.

**5:01** The nidus is now mobilized medially, and the plane between the cerebellum and the midbrain is explored, separating the AVM from the brainstem. This dissection proceeds until we reach the draining vein.

**5:25** The nidus is mobilized laterally, and the dissection proceeds now from medial to lateral in the deepest part of the cerebellomesencephalic fissure, separating the nidus from the midbrain until the lateral mesencephalic vein is reached.

**5:55** The draining vein is now blue and can be safely coagulated. It is coagulated as close as possible to the nidus in order to preserve normal venous drainage of the midbrain. The vein is now divided.

**6:24** The nidus is then removed.

**6:34** Here we can see the resection cavity and the final aspect.

**6:50** Here we can see postoperative CT scan showing the resection cavity and preservation of the brainstem.

**7:08** Postoperative angiogram confirmed surgical removal of the AVM, with no residual nidus.

**7:16** The patient recovered well from surgery. He was discharged home on postoperative day 6 with a baseline residual dysmetria. At 6-month follow-up, the patient recovered from his neurological symptoms and is now asymptomatic and neurologically intact.

## Author Contributions

Primary surgeon: Vieira. Assistant surgeon: Pereira. Editing and drafting the video and abstract: both authors. Reviewed submitted version of the work: Vieira. Approved the final version of the work on behalf of both authors: Vieira. Supervision: Vieira.
